# Failure of Effector Function of Human CD8^+^ T Cells in NOD/SCID/JAK3^−/−^ Immunodeficient Mice Transplanted with Human CD34^+^ Hematopoietic Stem Cells

**DOI:** 10.1371/journal.pone.0013109

**Published:** 2010-10-01

**Authors:** Yoshinori Sato, Hiroshi Takata, Naoki Kobayashi, Sayaka Nagata, Naomi Nakagata, Takamasa Ueno, Masafumi Takiguchi

**Affiliations:** 1 Center for AIDS Research, Kumamoto University, Kumamoto, Japan; 2 Division of Reproductive Engineering, Center for Animal Resources and Development, Kumamoto University, Kumamoto, Japan; New York University, United States of America

## Abstract

Humanized mice, which are generated by transplanting human CD34^+^ hematopoietic stem cells into immunodeficient mice, are expected to be useful for the research on human immune responses. It is reported that antigen-specific T cell responses occur in immunodeficient mice transplanted with both human fetal thymus/liver tissues and CD34^+^ fetal cells, but it remains unclear whether antigen-specific T cell responses occur in those transplanted with only human CD34^+^ hematopoietic stem cells (HSCs). Here we investigated the differentiation and function of human CD8^+^ T cells reconstituted in NOD/SCID/Jak3^−/−^ mice transplanted with human CD34^+^ HSCs (hNOK mice). Multicolor flow cytometric analysis demonstrated that human CD8^+^ T cells generated from the CD34^+^ HSCs comprised only 3 subtypes, i.e., CD27^high^CD28^+^CD45RA^+^CCR7^+^, CD27^+^CD28^+^CD45RA^−^CCR7^+^, and CD27^+^CD28^+^CD45RA^−^CCR7^−^ and had 3 phenotypes for 3 lytic molecules, i.e., perforin(Per)^−^granzymeA(GraA)^−^granzymeB(GraB)^−^, Per^−^GraA^+^GraB^−^, and Per^low^GraA^+^GraB^+^. These CD8^+^ T cells failed to produce IFN-γ and to proliferate after stimulation with alloantigens. These results indicate that the antigen-specific T cell response cannot be elicited in mice transplanted with only human CD34^+^ HSCs, because the T cells fail to develop normally in such mice.

## Introduction

Humanized mice are generated by transplanting human CD34^+^ hematopoietic stem cells (HSCs) into immunodeficient mice and are expected to become a useful tool in studies on human T cell immune responses, infectious diseases, preclinical testing of vaccines, and new therapeutic strategies. Previous studies showed long-term human T cell and B cell reconstitution in NOD/SCID/γc^null^ immunodeficient mice transplanted with human CD34^+^ HSCs (hNOG mice) [1]–[3]. Human IgM, IgG, and IgA were detectable in the serum of these mice; and class-switching of immunoglobulin in human cord blood (CB)-derived B cells properly occurred in the mice [4]–[7], indicating that the human B cells can develop from human CD34^+^ HSCs in the hNOG mice and are functionally competent to produce immunoglobulins in them. Furthermore, previous studies demonstrated that human CD4/CD8 double-positive and human CD4/CD8 single positive T cells were observed in the thymus of the hNOG mice and that the latter were found in the spleen and peripheral blood of these animals [3]–[5], [7], [8]. These human T cells expressed predominantly αβ T cell receptors in the thymus and the spleen of the recipient, whereas CD45RA^+^ naive T cells were identified in the spleen and the peripheral blood of the hNOG mice [4], [9], [10]. These results suggest the possibility that the human T cells respond to highly diverse molecules. Additionally, proliferation and IFN-γ expression of EBV-specific human CD8^+^ T cells have been demonstrated in hNOG mice and in Rag2^−/−^γc^−/−^ mice transplanted with human CD34^+^ HSCs after an EBV infection [8], [11]. In contrast, high-dose injection of EBV caused a fatal lymphoproliferative disorder in the hNOG mice, whereas lower-dose injection induces an apparently asymptomatic persistent infection [11]. These findings suggest that the human T cell responses were not able to control the replication of EBV in the hNOG mice.

On the other hand, antigen-specific T cell immune responses had definitely been shown in humanized NOD/SCID mice established by transplanting human fetal thymus/liver tissues and CD34^+^ fetal liver cells into them (BLT mice) [12]–[16]. However, there are no studies providing reliable evidence as to whether or not antigen-specific T cell responses are induced in humanized mice established by transplanting only human CD34^+^ HSCs into immunodeficient mice.

The phenotypic analysis of human T cells in humanized mice is useful to clarify their differentiation and effector function, because the phenotypic classification of human T cells reflects their differentiation and effector function. Previous studies demonstrated that human CD8^+^ T cells change the expression levels of co-stimulatory molecules (CD27, CD28, and CD45RA) [17]–[19] and chemokine receptor CCR7 on their surface according to their differentiation and maturation [20], [21]. The phenotypic analysis of human CD8^+^ T cells showed that CD27^high^CD28^+^CD45RA^+^CCR7^+^, CD27^+^CD28^+^CD45RA^−^CCR7^+^, CD27^+^CD28^+^CD45RA^−^CCR7^−^, CD27^low^CD28^−^CD45RA^+/−^CCR7^−^, and CD27^−^CD28^−^CD45RA^+/−^CCR7^−^ have characteristics of naive, central memory, early effector memory, late effector memory, and effector CD8^+^ T cells, respectively [22], [23]. Moreover, human CD8^+^ T cells express 3 key cytolytic effector molecules, i.e., perforin (Per), granzyme A (GraA), and granzyme B (GraB) in response to their differentiation [24]. Five subpopulations of human CD8^+^ T cells defined by these cytolytic molecules exist and appear sequentially during CD8^+^ T cell differentiation: Per^−^GraA^−^GraB^−^, Per^−^GraA^+^GraB^−^, Per^low^GraA^+^GraB^−^, Per^low^GraA^+^GraB^+^, and Per^high^GraA^+^GraB^+^ [25]. Thus, the functional subsets of human CD8^+^ T cells can be identified by the phenotypic classification and the expression of these 3 cytolytic molecules [25]. By using these classifications of human CD8^+^ T cells, the differentiation and function of human CD8^+^ T cells reconstituted in humanized mice can be clarified in detail. A previous study showed that the CD45RA^+^CCR7^−^ subset were detected in hNOG mice [10], suggesting the possibility that human CD8^+^ T cells can be differentiated in the mice. However, since this study did not demonstrated the existence of effector subset, CD27^−^CD28^−^CD45RA^+/−^CCR7^−^, it still remains unclear if human CD8^+^ T cells can be differentiated into effector cells in hNOG mice.

In the present study, to clarify antigen-specific human CD8^+^ T cell responses in immunodeficient mice transplanted with only human CD34^+^ HSCs, we established NOD/SCID/JAK3^−/−^ (NOK) mice transplanted with human CD34^+^ HSCs (hNOK mice) and investigated the differentiation and function of human CD8^+^ T cells reconstituted in these mice. Especially, we focused on performing phenotypic classification based on the expression of the above-mentioned effector molecules and the alloreactivity of human CD8^+^ T cells reconstituted in the mice.

## Results

### Reconstitution of human T cells in hNOK mice

**Figure 1:**
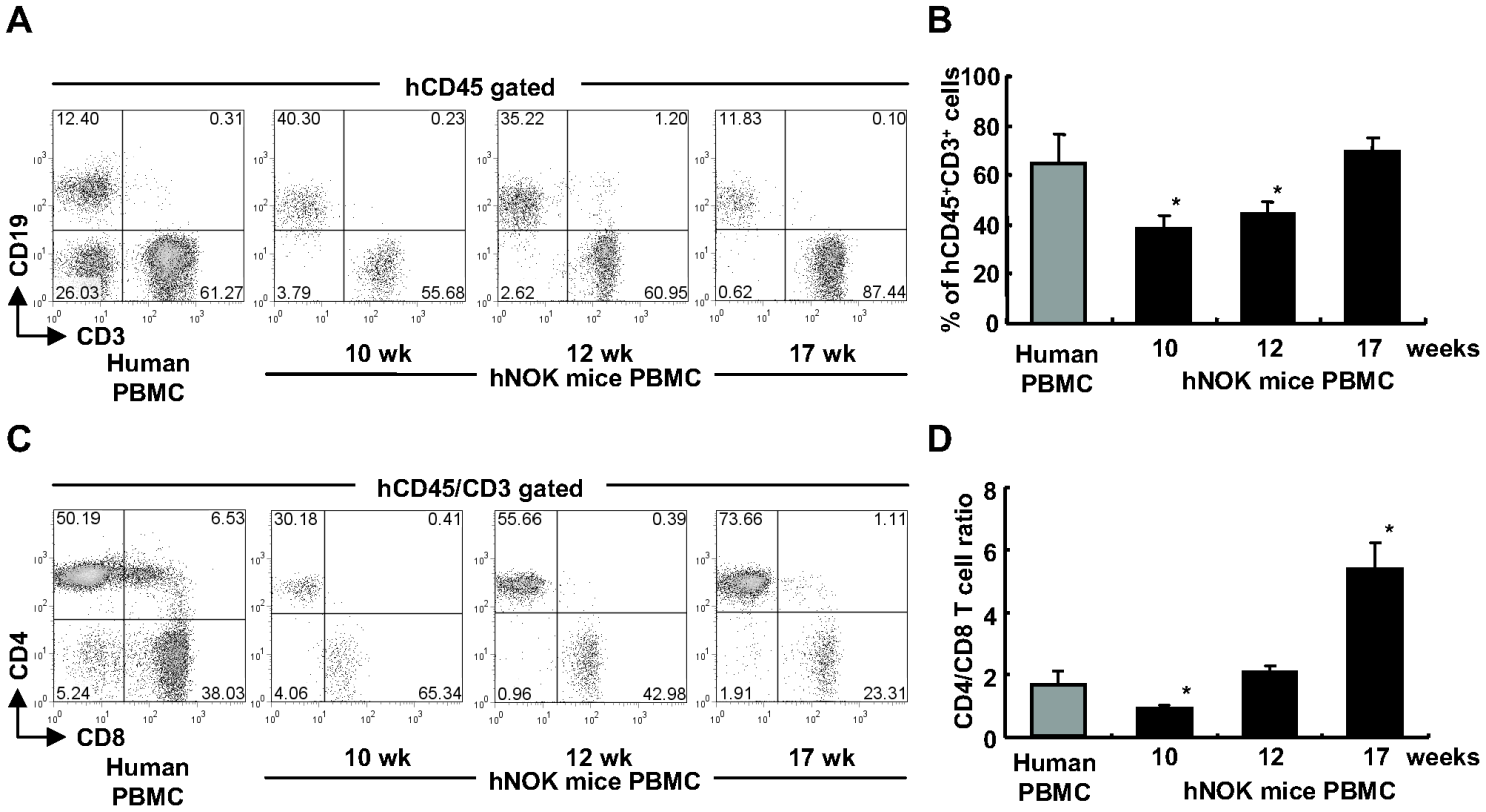
Flow cytometric analysis of human T cells reconstituted in hNOK mice. (A) A representative result for hCD45^+^CD3^+^ T cells and hCD45^+^CD19^+^ B cells among PBMC from hNOK mice at 10, 12, and 17 weeks after the transplantation. (B) Summarized results showing hCD45^+^CD3^+^ T cell proportion in PBMC from hNOK mice (n = 20) at 10, 12 and 17 weeks. (C) A representative result for human CD4^+^ and CD8^+^ T cell proportions in PBMC from hNOK at 10, 12 and 17 weeks after the transplantation. (D) Summarized results showing human CD4/CD8 T cell ratio for PBMC from hNOK mice at 10, 12, and 17 weeks. Human PBMC from healthy adult individuals (n = 4) are shown as a control. Bar graph data are shown as the mean ± SEM of 7 independent experiments. *, *p<0.05*, human PBMC vs. hNOK mouse PBMC.

hNOK mice were established by transplanting human CD34^+^ HSCs isolated from human CB into the liver of newborn NOK mice. In order to investigate the reconstitution of the human immune system in these mice, we obtained PBMC from the mice at 10, 12 and 17 weeks after the transplantation and then analyzed them by using flow cytometry for detecting human T and B cells. As shown in Table 1, the cells expressing common human leukocyte antigen CD45 (hCD45^+^) were found in all hNOK mice at 10 weeks after the transplantation (n = 31). Among the hCD45^+^ cells, human B cells, identified by the expression of CD19, were observed in all hNOK mice, whereas human T cells, identified by the expression of CD3, were observed in approximately 60% of the mice (Table 1). In the mice carrying human T cells, the proportion of human T cells gradually increased from 10 to 17 weeks after the transplantation and reached a level at 17 weeks similar to the proportion of CD8^+^ T cells in the human adult PBMC population (Figure 1A and 1B). The human T cells obtained during the period of 10-17 weeks post transplantation included both human CD4^+^ and CD8^+^ T cells (Figure 1C), though the ratio of human CD4/CD8 T cells gradually increased from the 10 to 17 weeks (Figure 1D). These results show that human CD8^+^ and CD4^+^ T cells were generated and maintained in the mice.

**Table 1:** Proportion of human immune cells in hNOK mice.

		% nucleated cells					% nucleated cells		
Cord blood		hCD45 gated		Cord blood		hCD45 gated	
Mouse No.		hCD45^+^	CD3^+^	CD19^+^	Mouse No.		hCD45^+^	CD3^+^	CD19^+^
Donor 1	1	12.8	0.0	54.6	Donor 4	1	62.1	46.7	48.3
	2	32.7	32.3	61.8		2	37.4	37.0	58.8
	3	55.1	55.7	40.3		3	57.4	23.3	67.8
	4	59.1	0.0	89.3		4	47.3	45.0	49.2
	5	5.1	0.5	94.9		5	21.4	0.0	91.0
	6	34.2	38.0	57.5		6	37.1	33.7	57.5
Donor 2	1	45.8	0.1	67.9	Donor 5	1	32.1	0.2	95.0
	2	35.1	13.1	57.9		2	10.3	0.0	92.9
	3	55.6	49.7	40.6		3	64.8	30.2	67.3
	4	19.3	0.0	65.5		4	44.2	0.0	96.5
	5	69.4	39.5	48.4		5	3.5	0.0	81.0
Donor 3	1	5.8	0.4	76.2	Donor 6	1	6.6	5.2	81.0
	2	18.9	47.0	43.4		2	7.7	81.3	13.2
	3	37.6	84.1	11.7	Donor 7	1	10.5	34.1	60.7
	4	10.7	0.2	79.7		2	50.3	42.2	54.0
	5	58.4	73.6	22.6					

For generation of hNOK mice, human CD34^+^ cells derived from 7 cord blood samples were transplanted into newborn NOK mice. PBMC of the hNOK mice were analyzed for the engraftment of human immune cells at 10 weeks after the transplantation (n = 31).

### Phenotypic analysis of human T cells reconstituted in hNOK mice

**Figure 2:**
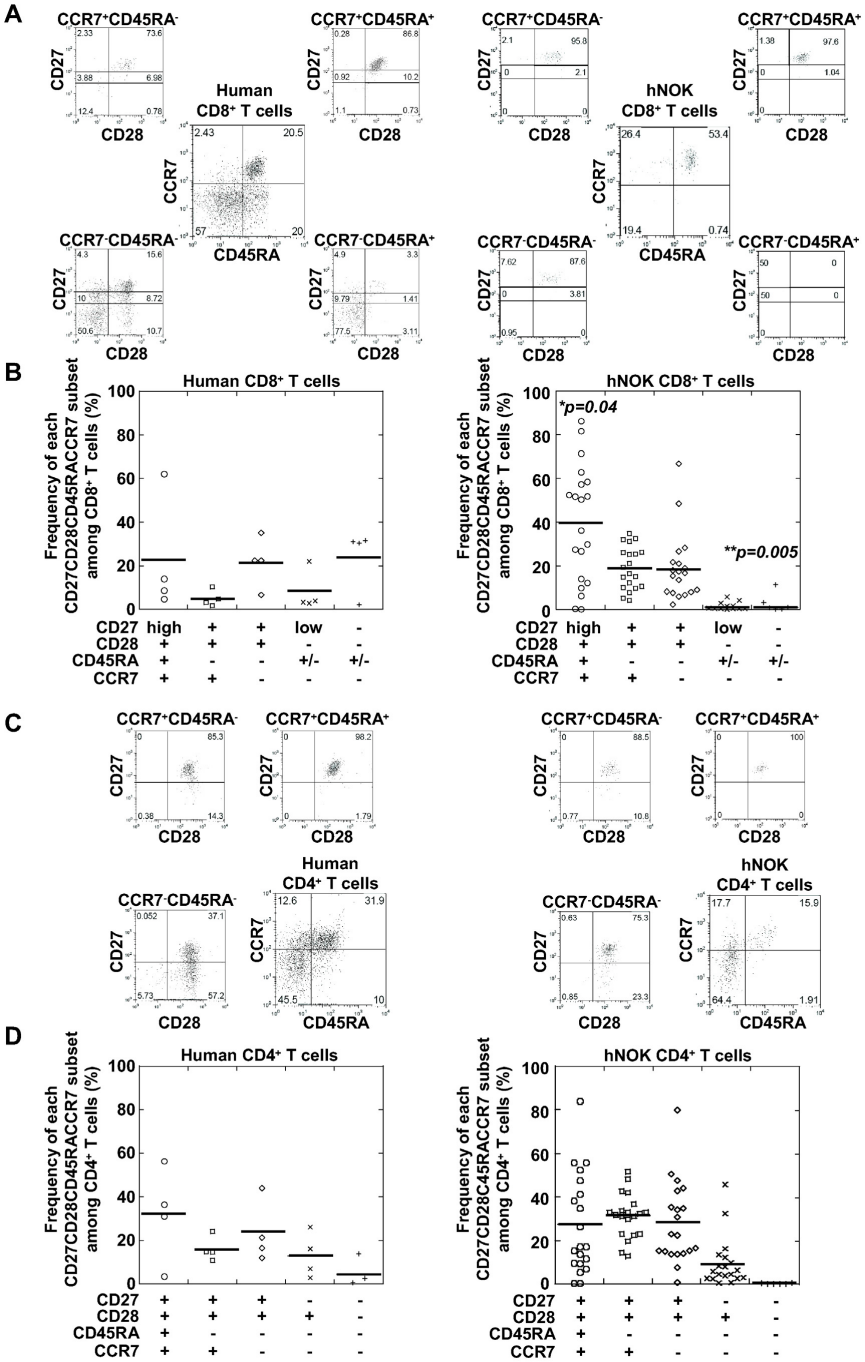
Phenotypic classification of human CD8^+^ T cells reconstituted in hNOK mice. Human T cells among PBMC from hNOK mice at 17 weeks after the transplantation were analyzed for the expression of the following cell-surface markers: CD4, CD8, CD27, CD28, CD45RA, and CCR7. (A) A representative result of 5-color flow cytometric analysis of the human CD8^+^ T cell population among PBMC from a hNOK mouse (right data) and an adult human (left data). (B) Summarized results showing frequency of subsets among the human CD8^+^ T cells isolated from the PBMC from hNOK mice (n = 20, right data) and human adult individuals (n = 4, left data). (C) A representative result of 5-color flow cytometric analysis of human CD4^+^ T cell population in PBMC from hNOK mice (right data) and a human adult individual (left data). (D) Summarized result showing human CD4^+^ T cell proportion in PBMC from hNOK mice (n = 20, right data) and human adult individuals (n = 4, left data). Each symbol represents 1 mouse; the mean value is shown as a horizontal solid line. *, *p<0.05*; **, *p<0.01*, human PBMC vs. hNOK mice PBMC.

Human peripheral CD8^+^ T cells are classified into the following 5 major populations based on their expression of 4 cell-surface markers: CD27^high^CD28^+^CD45RA^+^CCR7^+^ (naive subset), CD27^+^CD28^+^CD45RA^−^CCR7^+^ (central memory subset), CD27^+^CD28^+^CD45RA^−^CCR7^−^ (early effector memory subset), CD27^low^CD28^−^CD45RA^+/−^CCR7^−^ (late effector memory subset), and CD27^−^CD28^−^CD45RA^+/−^CCR7^−^ (effector subset) [22], [23]; whereas human peripheral CD4^+^ T cells are also classified into 5 major populations by the same 4 cell-surface markers, i.e., CD27^+^CD28^+^CD45RA^+^CCR7^+^ (naive subset), CD27^+^CD28^+^CD45RA^−^CCR7^+^ (central memory subset), CD27^+^CD28^+^CD45RA^−^CCR7^−^ (Th0 effector memory subset), CD27^-^CD28^+^CD45RA^−^CCR7^−^ (Th1/2 effector memory subset), and CD27^−^CD28^−^CD45RA^−^CCR7^−^ (effector subset) [26]. To examine the differentiation of human T cells reconstituted in the hNOK mice, we analyzed the phenotype of human T cells among PBMC from the mice at 17 weeks after the transplantation and that of T cells among PBMC from adult humans for comparison. Representative results for an individual hNOK mouse and an adult human are shown in Figure 2A; and a summary of the findings, in Figure 2B. The reconstituted human CD8^+^ T cell population included naive (39.7±26.9%), central memory (18.9±9.7%), and early effector memory (18.4±15.9%) subsets. Late effector memory (0.9±1.6%) and effector (0.8±2.6%) subsets were hardly detected in the mice. These results suggest that the human CD8^+^ T cells did not differentiate into late effector or effector human T cells in the mice.

The phenotypic analysis of the reconstituted human CD4^+^ T cell population in the PBMC from the mice revealed that it included naive (27.5±23.0%), central memory (31.2±10.1%), Th0 effector memory (28.1±18.8%), and Th1/2 effector memory (9.0±11.4%) subsets. The effector (0.06±0.15%) subset was hardly detected, suggesting that the human CD4^+^ T cells did not differentiate into effector T cells in these mice.

### Expression of 3 cytolytic effector molecules in reconstituted human CD8^+^ T cells

**Figure 3:**
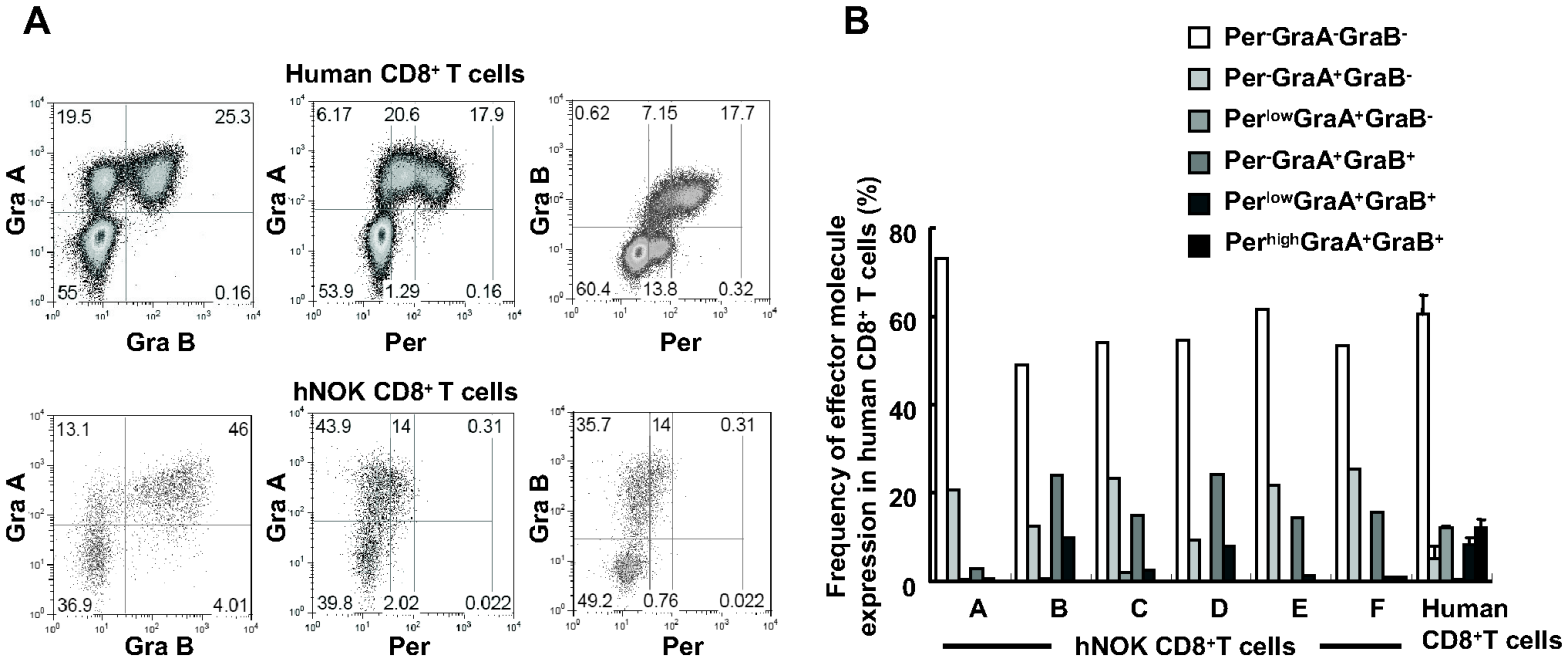
Expression of 3 cytolytic effector molecules of human CD8^+^ T cells reconstituted in hNOK mice. Human CD8^+^ T cells among splenocytes of hNOK mice were analyzed for the expression of 3 cytolytic effector molecules: Per, Gra A, and Gra B. (A) A representative result showing co-expression of 3 cytolytic effector molecules in human CD8^+^ T cells from a hNOK mouse (lower data) and a human adult individual (upper data). (B) Summarized result for the expression of 3 the cytolytic effector molecules in human CD8^+^ T cells from hNOK mice (n = 6) and human adult individuals (n = 3).

Human peripheral CD8^+^ T cells are classified into the following 5 major populations based on their expression levels of 3 effector molecules: Per^−^GraA^−^GraB^−^, Per^−^GraA^+^GraB^−^, Per^low^GraA^+^GraB^−^, Per^low^GraA^+^GraB^+^, and Per^high^GraA^+^GraB^+^ [25]. To investigate the effector function of the human CD8^+^ T cells reconstituted in the hNOK mice, we examined the expression of these effector molecules in human CD8^+^ T cells among splenocytes from the mice. A representative result and summary of the analysis for 6 hNOK mice as well as for PBMC from 3 adult humans are shown in Figure 3A and 3B, respectively. The human CD8^+^ T cells included Per^−^GraA^−^GraB^−^, Per^−^GraA^+^GraB^−^, and Per^low^GraA^+^GraB^+^ cells. But they did not include Per^low^GraA^+^GraB^−^ or Per^high^GraA^+^GraB^+^ ones, though the latter were found among human adult PBMC. These results indicate that the reconstituted human CD8^+^ T cells did not have effector function in the mice and that the human CD8^+^ T cells did not differentiate into effector cells in the mice.

### Ability of reconstituted human CD8^+^ T cells to produce cytokines

**Figure 4:**
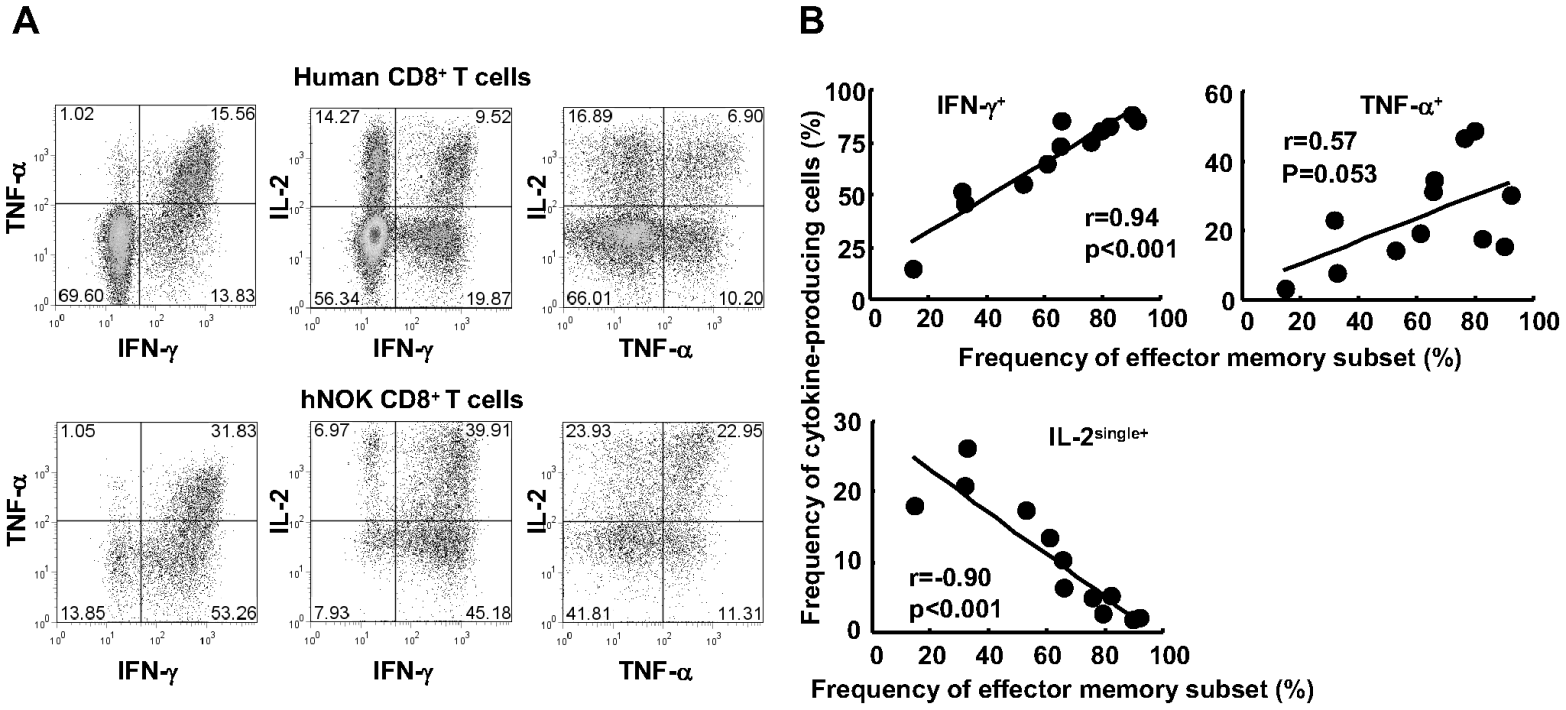
Cytokine production of human CD8^+^ T cells reconstituted in hNOK mice. Splenocytes from hNOK mice were cultured with PMA and ionomycin for 6 hours and then the production of IFN-γ, TNF-α, and IL-2 cytokines by human CD8^+^ T cells was measured by flow cytometry. (A) A representative result for cytokine production by human CD8^+^ T cells from a hNOK mouse (lower data) and a human adult individual (upper data). (B) Correlation between frequency of IFN-γ-, TNF-α-, and IL-2^+^IFN-γ^−^TNF-α^−^ (IL-2^single+^)-producing CD8^+^ T cells and that of effector memory (CD27^+^CD28^+^CCR7^−^CD45RA^−^ plus CD27^low^CD28^−^CD45RA^+/−^CCR7^−^) CD8^+^ T cell subset (n = 12).

Human CD8^+^ T cells producing IL-2 but not IFN-γ are mainly found in naive and central memory CD8^+^ T cell populations, whereas IFN-γ and TNF-α are mainly produced by effector memory and effector CD8^+^ T cells among human PBMC [22], [27]. To investigate the ability of the human CD8^+^ T cells reconstituted in the hNOK mice to produce these cytokines, we stimulated splenocytes isolated from these mice with PMA and ionomycin *in vitro* and measured the production of the above cytokines. A representative result and summary of the analysis for 12 hNOK mice are given in Figure 4A and 4B, respectively. The human CD8^+^ T cells reconstituted in the mice produced IFN-γ, TNF-α, and IL-2 (Figure 4A). The frequency of IFN-γ-producing CD8^+^ T cells positively correlated with that of the effector memory (CD27^+^CD28^+^CD45RA^−^CCR7^−^ plus CD27^low^CD28^−^CD45RA^+/−^CCR7^−^) subset, whereas the frequency of IL-2^+^IFN-γ^−^TNF-α^−^-producing CD8^+^ T cells negatively correlated with that of effector memory subset (Figure 4B). These results indicate that the human CD8^+^ T cells had the ability to produce these cytokines in the mice.

### Alloreactivity of human CD8^+^ T cells reconstituted in hNOK mice

**Figure 5:**
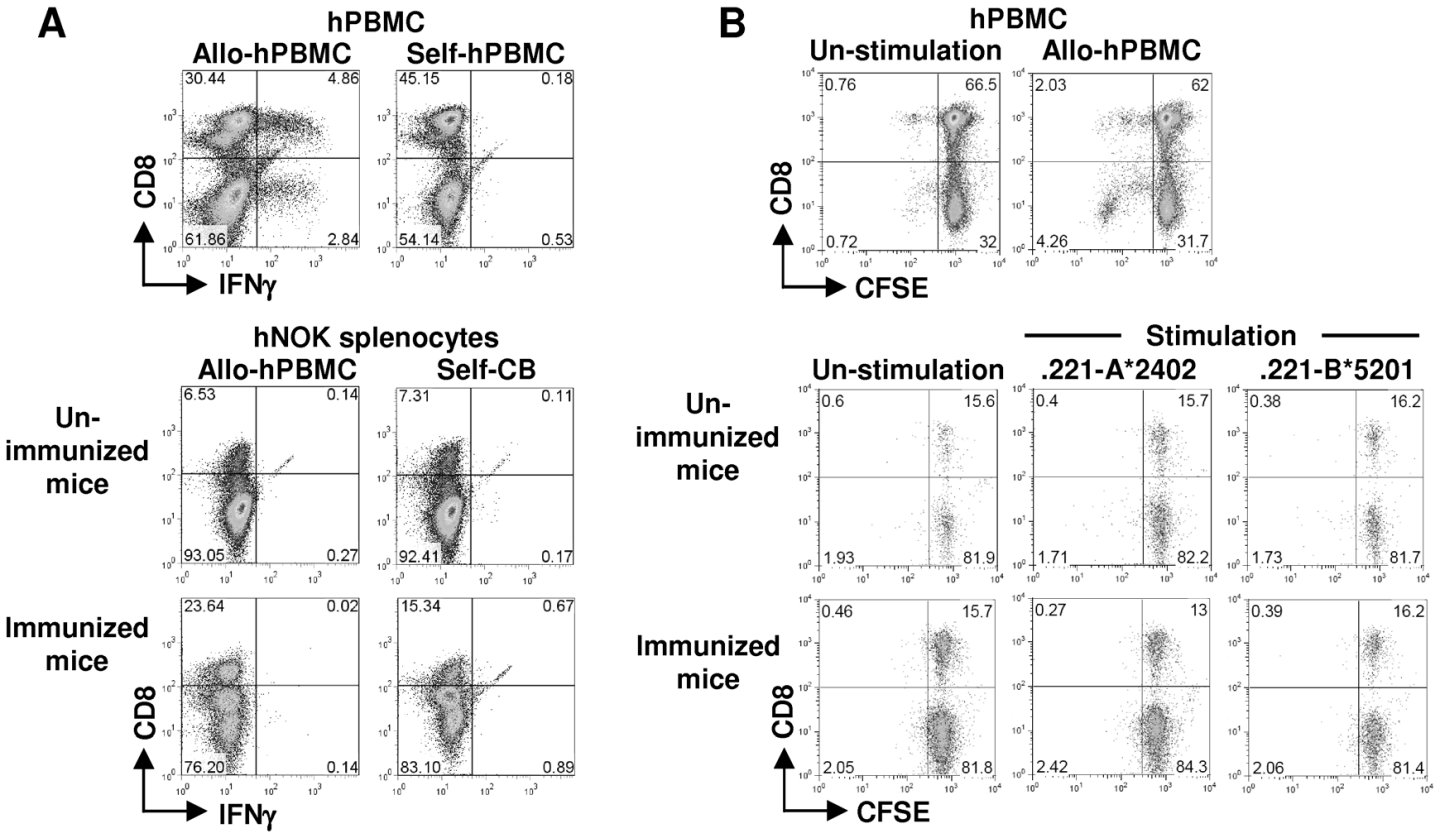
Alloreactivity of human CD8^+^ T cells reconstituted in hNOK mice. The hNOK mice were immunized for 31 days with irradiated human PBMC (allo-hPBMC) from a healthy donor with HLA-A*2402/A*2402, HLA-B*5201/B*5901, and HLA-DRB1*1502/DRB1*0405 (immunized mice) or PBS (un-immunized mice). The splenocytes of each hNOK mice were harvested, and the cells were stained by using anti-human CD45, anti-human CD3, and anti-human CD8 mAbs. (A) Human adult PBMC from a healthy donor with HLA-A*2601/A*2403, HLA-B*3501/B*5101, and HLA-DRB1*0405/DRB1*0405 were cultured with the allo-hPBMC or the self-hPBMC for 7 days *in vitro*, and a representative result showing the IFN-γ production by the human CD8^+^ T cells and the human CD8^−^ T cells is shown as a control (upper data). The splenocytes from the immunized hNOK mice were cultured with the allo-hPBMC or self-CB for 7 days *in vitro*, and a representative result for IFN-γ production by the human CD8^+^ T cells and the human CD8^−^ T cells is shown (lower data). (B) Splenocytes from the immunized hNOK mice were cultured with irradiated 721.221 cells expressing HLA-A*2402 (.221-A*2402) or irradiated 721.221 expressing HLA-B*5201 (.221-B*5201) for 3 days *in vitro*, and a representative result for the proliferation of the human CD8^+^ T cells or the human CD8^−^ T cells is shown. One representative experiment from 3 experiments is shown.

It is well known that 0.1–10% of human T-cell repertoire react with alloantigens compared with a frequency of <1/100,000 for foreign antigens in humans [28], [29]. We investigated alloreactivity of human CD8^+^ T cells reconstituted in hNOK mice to clarify the responsiveness of antigen-specific human CD8^+^ T cells. We immunized hNOK mice with irradiated human PBMC from a healthy donor with HLA-A*2402/A*2402, B*5201/B*5901, and DRB1*1502/DRB1*0405 (allo-PBMC) (n = 4) and analyzed the phenotype of human CD8^+^ T cells reconstituted in the mice on 7 days or 31 days after the PBMC injection. Flow cytometric analysis demonstrated that CD27^−^CD28^−^CD45RA^+/−^CCR7^−^ effector CD8^+^ T cell subsets and Per^high^GraA^+^GraB^+^ cells were not induced in the mice on 7 days and 31 days after the PBMC injection (Figures S1A and S1B). We further analyzed the ability of the human CD8^+^ T cells to produce IFN-γ after allo-PBMC stimulation. The splenocytes from the mice obtained at 31 days after injection of the irradiated PBMC into the mice were cultured with the irradiated PBMC *in vitro* for 7 days, and then the ability of the human CD8^+^ T cells to produce IFN-γ was measured after stimulation with the irradiated PBMC. As shown in Figure 5A, the human CD8^+^ T cells and the CD8^−^ T cells (mostly CD4^+^ T cells) reconstituted in the mice did not produce IFN-γ, suggesting that both CD8^+^ T cells and CD4^+^ T cells could not recognize alloantigen. In addition, we analyzed the ability of the human CD8^+^ T cells to proliferate after stimulation with alloantigens. The splenocytes of the mice on 31 days were labeled with CFSE (carboxyfluorescein diacetate N-succinimidyl ester); and the cells were cultured for 3 days with irradiated 721.221 cells expressing HLA-A*2402 (.221-A*2402) or irradiated 721.221 expressing HLA-B*5201 (.221-B*5201) *in vitro*. As shown in Figure 5B, the human CD8^+^ T cells and the CD8^−^ T cells (mostly CD4^+^ T cells) reconstituted in the mice did not proliferate in response to.221-A*2402 or. 221-B*5201 cells. In addition, they did not proliferate in response to splenocytes of C57BL/6(H-2^b^) or Balb/c (H-2^d^) mice (data not shown). These results together indicate that the human CD8^+^ T cells could not recognize alloantigens in the mice.

## Discussion

It had remained unknown whether antigen-specific human T cell responses can be elicited in immunodeficient mice transplanted with only human CD34^+^ HSCs. In the present study, we analyzed the differentiation of the reconstituted human T cells and the function of the human CD8^+^ T cells stimulated by alloantigens in hNOK mice. Because human T cells express 4 cell-surface markers (CD27, CD28, CD45RA, and CCR7) and 3 cytolytic molecules (GraA, GraB, and Per) at different levels according to their stage of differentiation and maturation [22], [23], [25], the analysis of phenotype and effector molecule expression in the reconstituted human T cells is useful to clarify their differentiation status and effector function. We demonstrated that the CD27^low^CD28^−^CD45RA^+/−^CCR7^−^ (late effector memory) and the CD27^−^CD28^−^CD45RA^+/−^CCR7^−^ (effector) subsets did not exist among the human CD8^+^ T cells from the hNOK mice. In addition, the reconstituted human CD8^+^ T cells did not include the Per^high^GraA^+^GraB^+^ (effector) cells. These results suggest that the reconstituted human CD8^+^ T cells did not have their effector function *in vivo*. On the other hand, the reconstituted human CD8^+^ T cells included the CD27^+^CD28^+^CD45RA^−^CCR7^−^ (early effector memory) subsets in the hNOK mice, suggesting that the human CD8^+^ T cells could differentiate into memory T cells in the hNOK mice in response to some stimulation. Although early effector memory CD8^+^ T cells among the human adult CD8^+^ T cell population include Per^low^GraA^+^GraB^−^ cells [25], the reconstituted human CD8^+^ T cells did not include the Per^low^GraA^+^GraB^−^ ones. This finding suggests that some population of early effector memory CD8^+^ T cells in the mice were defective and could not express Per in the course of their differentiation. These results further suggest that the human CD8^+^ T cells did not have their effector function in the hNOK mice. On the other hand, the reconstituted human CD8^+^ T cells showed the ability to produce cytokines similar to that of human adult CD8^+^ T cells.

The linear differentiation model supports the idea that the naive T cells differentiate directly into effector cells during the acute phase of the response and that following contraction in the numbers of the effector cells at the end of the primary response, effector memory and central memory T cells become detectable [30]. However, the human CD8^+^ T cells reconstituted in the hNOK mice showed the lack of Per expression and Per^low^GraA^+^GraB^−^ subset, implying that the cells could not differentiate into effector cells and subsequently differentiate into memory cells. The results of phenotypic analysis and effector molecule expression of the reconstituted human CD8^+^ T cells in the hNOK mice support the idea that the human CD8^+^ T cells asymmetrically divided into long-lived memory cells [30], [31].

A previous study demonstrated the presence of alloantigen-specific cytotoxic human T cell clones in hNOG mice [5]. However, the data from that study do not exactly reflect T cell responses *in vivo*; because alloantigen-specific human CD8^+^ T cell clones were established from the spleen cells of the mice cultured with allogeneic target cells *in vitro*. The frequency of the human alloreactive T cell repertoire was evaluated to reflect between 0.1% and 10% of the total T cell population, whereas that of T cells specific for foreign antigen is <1/100,000 [28], [29]. Since the reconstituted human CD8^+^ T cells did not respond to alloantigen in the hNOK mice, these reconstituted human CD8^+^ T cells may not have a foreign antigen-specific function *in vivo*. This result implies that the reconstituted human CD8^+^ T cells did not have the appropriate T cell receptor (TCR) repertoire against foreign antigens, because the human CD8^+^ T cells were educated by mouse MHC-peptide complexes in the thymus. CD8 co-receptors bind with a nonpolymorphic region within the α3 domain of class I MHC proteins, and their binding has species specificity [32]–[36]. Considering that human CD8 molecules fail to bind to murine MHC, the human CD8^+^ T cells in immunodeficient mice transplanted with only human CD34^+^ HSCs are not educated in the murine thymus.

There are several reports that antigen-specific responses are detectable in BLT mice infected with EBV or HIV [12], [14], [16]. Furthermore, the BLT mice produce high levels of human IgM and IgG antibodies and mediate strong immune responses *in vivo*, as demonstrated by skin xenograft rejection [13]. These results suggest that the reconstituted human T cells educated in the human thymus can have appropriate antigen-specific function in mice. A recent study demonstrated that the human CD8^+^ T cells reconstituted in HLA transgenic NOG mice transplanted with only human CD34^+^ HSCs expressed IFN-γ production and EBV-specific cytolytic activity in response to an EBV infection [37], [38], suggesting that the reconstituted human T cells educated by human HLA class I molecules on mouse thymus cells have appropriate effector function in the mice.

In the present study, we demonstrated impaired differentiation of human CD8^+^ T cells in the hNOK mice established with only human CD34^+^ T cells and lack of ability to induce the effector function. Previous studies of hNOG mice showed very weak immune responses to viral antigens [8], [9], [11]. Thus both hNOK and hNOG mice seems to have no or weak ability in HLA-restricted T cell responses although it is unclear whether some differences exist in human T cell function between 2 humanized mice. HLA transgenic immunodeficient mice are expected to be useful for the generation of a good mouse model having human immunity.

## Materials and Methods

### Establishment of humanized mice

The NOK mouse strain was established by backcrossing JAK3^−/−^ mice with the NOD.Cg-Prkdcscid strain for 10 generations. The NOK mice were maintained under specific pathogen-free conditions in the Center for Animal Resources and Development. Animal experiments were conducted according to the Regulation for Animal Experiments in Kumamoto University (Approval ID, C22-168: Analysis of immune responses against viral diseases by using humanized and HLA transgenic mice). Human CB was purchased from Riken Cell Bank (Tsukuba, Japan). Human CD34^+^ cells were isolated from human CB by using a Direct CD34 Progenitor Cell Isolation Kit and an MS Column (Miltenyi Biotec, Gladbach, Germany). The usual purity of the human CD34^+^ cells was approximately 95%. The hNOK mice were generated by injecting the isolated human CD34^+^ cells (5×10^4^ cells/mouse) into the liver of newborn NOK mice.

### Blood samples

Human blood samples were taken from healthy adult individuals. Peripheral blood mononuclear cells (PBMC) were isolated from the blood by using Ficoll-Paque PLUS (GE Healthcare, Uppsala, Sweden).

### Cell lines

721.221 cells are human B cell lines lacking HLA class I but not HLA class II [39]. 721.221 cells expressing HLA-A*2402 (.221-A*2402) and those expressing HLA-B*5201 (.221-B*5201) were generated by transfecting of HLA-A*2402 and HLA-B*5201 genes into 721.221 cells, respectively.

### Flow cytometric analysis

Human leukocytes reconstituted in hNOK mice were stained with various combinations of monoclonal antibodies (mAbs): FITC-labeled anti-mouse CD45 (mCD45), PECy7-labeled human CD45 (hCD45), allophycocyanin-labeled anti-CD4, allophycocyanin, Cy7-labeled anti-CD4, AmCyan-labeled anti-CD8, Pacific blue-labeled anti-CD8, PE-labeled anti-CD19, FITC-labeled anti-Per, PE-labeled anti-GraA, Alexa647-labeled anti-GraB, PE-Cy7-labeled anti-CCR7, FITC-labeled anti-CD45RA, PE-labeled anti-CD28, allophycocyanin-Cy7-labeled anti-CD27, FITC-labeled IFN-γ and PE-Cy7-labeled TNF-α mAbs, all were purchased from BD Biosciences (San Diego, CA). ECD-labeled anti-CD3 mAb was obtained from Beckman Coulter (Fullerton, CA); and allophycocyanin-labeled IL-2 mAb, from e-bioscience (San Diego, CA). To analyze the phenotype of human T cells reconstituted in the hNOK mice, we first stained the human T cells with anti-CCR7 mAb for 30 min at room temperature, and subsequently with specific antibody against surface markers at 4°C for 30 min. The cells were washed twice with PBS containing 10% fetal calf serum (FCS; Sigma-Aldrich, St. Louis, MO). To analyze the intracellular expression of IFN-γ, Per, GraA, and GraB, we fixed cells with 4% paraformaldehyde PBS at 4°C for 20 min, and then made them permeable by incubating them at 4°C for 10 min in PBS containing 0.1% saponin (Sigma-Aldrich) and 20% FCS (permeabilizing buffer). The cells were then stained with anti-IFN-γ, anti-Per, anti-GraA, and anti-GraB mAbs at 4°C for 20 min. Finally, the stained cells were washed 3 times in the permeabilizing buffer at 4°C. The stained cells were analyzed by using a FACSCant II flow cytometer (BD Biosciences, San Jose, CA). All flow cytometric data were analyzed by using FlowJo software (Tree Star, Inc, Ashland, OR).

### Cytokine production of human CD8^+^ T cells stimulated with PMA and ionomycin

The splenocytes of hNOK mice were cultured at a density of 1×10^6^ cells in 96- well plates with phorbol 12-myristate 13-acetate (PMA) (10 ng/ml) and ionomycin (1 ng/ml). Two hours later, brefeldin A (10 ng/ml) was added to each well. After a further 4-hour incubation, the splenocytes were stained with Allophycocyanin-labeled anti-IL-2, FITC-labeled anti-IFN-γ, and PE-Cy7-labeled anti-TNF mAbs, and the stained cells were analyzed on the FACSCant II flow cytometer.

### Assay for alloreactivity of human CD8^+^ T cells

PBMC (5×10^6^/mouse) from a healthy donor with HLA types HLA-A*2402/A*2402, B*5201/B*5901, and DRB1*1502/DRB1*0405 were irradiated and then intraperitoneally injected into hNOK mice. At 4 weeks after the injection, PBMC and splenocytes of the hNOK mice were harvested and analyzed for their phenotype by using the FACSCant II. To investigate IFN-γ expression of human T cells against alloantigen, we cultured the splenocytes for 6 hours at a density of 1×10^6^ cells in 96-well plates with the irradiated PBMC or irradiated self-CB. Then the cultured cells were stained with anti-CD3 mAb, anti-CD8 mAb, and anti-IFN-γ mAb. For proliferation assays, carboxyfluorescein diacetate N-succinimidyl ester (CFSE, Molecular Probes, Willow Creek Road Eugene, OR) for a final concentration of 0.5 µM was added to the cell suspension. After a 15-min incubation, the excess CFSE was removed by washing with 5% FCS/PBS; and then the cells were resuspended in RPMI1640 containing 10% FCS. The CFSE-labeled cells were cultured for 3 days with 721.221 cells expressing HLA-A*2402 or HLA-B*5201 and then were analyzed on the FACSCant II.

### Statistical analysis

Results shown as bar graphs were expressed as the means ± S.E.M. One-way ANOVA followed by Dunnet's test was used for multiple comparisons. Differences were considered to be statistically significant when the *p*-value was less than 0.05.

## Supporting Information

Figure S1 Phenotypic analysis of reconstituted human CD8^+^ T cells stimulated with alloantigen in hNOK mice hNOK mice were immunized for 31 days with irradiated human PBMC from a healthy donor with HLA-A*2402/A*2402, HLA-B*5201/B*5901, and HLA-DRB1*1502/DRB1*0405. Then the phenotype of human CD8^+^ T cells among PBMC from the hNOK mice was analyzed on 0, 7, and 31 days after the immunization. Splenocytes from the same mice were examined at day 31. (A) Representative results of 5-color flow cytometric analysis of CCR7CD45RACD27CD28 subsets in human CD8^+^ T cell population of PBMC and splenocytes are shown. (B) Representative results for Per, GraA, and GraB expression by the human CD8^+^ T cells among splenocytes from the hNOK mice are shown.
(1.97 MB TIF)
