# Supplementary figures and images for: Failure of Effector Function of Human CD8+ T Cells in NOD/SCID/JAK3−/− Immunodeficient Mice Transplanted with Human CD34+ Hematopoietic Stem Cells
Source: PLoS One. 2010 Oct 1;5(10):e13109. doi: 10.1371/journal.pone.0013109 (PMC2948507; doi:10.1371/journal.pone.0013109)

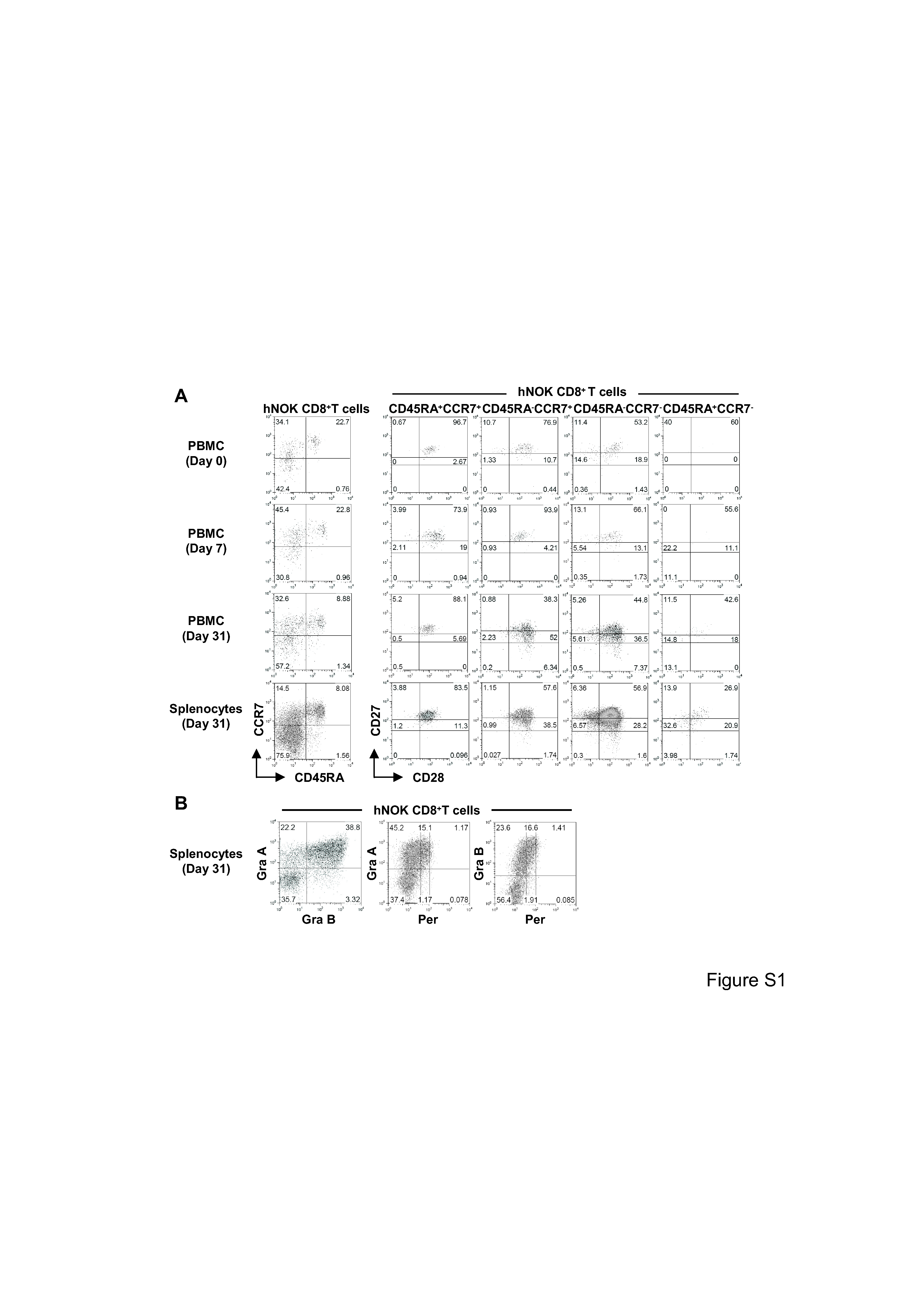

Supplement: Figure S1 — Phenotypic analysis of reconstituted human CD8+ T cells stimulated with alloantigen in hNOK mice hNOK mice were immunized for 31 days with irradiated human PBMC from a healthy donor with HLA-A*2402/A*2402, HLA-B*5201/B*5901, and HLA-DRB1*1502/DRB1*0405. Then the phenotype of human CD8+ T cells among PBMC from the hNOK mice was analyzed on 0, 7, and 31 days after the immunization. Splenocytes from the same mice were examined at day 31. (A) Representative results of 5-color flow cytometric analysis of CCR7CD45RACD27CD28 subsets in human CD8+ T cell population of PBMC and splenocytes are shown. (B) Representative results for Per, GraA, and GraB expression by the human CD8+ T cells among splenocytes from the hNOK mice are shown. (1.97 MB TIF) [file pone.0013109.s001.tif]
